# Epidemiology of Noble Pen Shell (*Pinna nobilis* L. 1758) Mass Mortality Events in Adriatic Sea Is Characterised with Rapid Spreading and Acute Disease Progression

**DOI:** 10.3390/pathogens9100776

**Published:** 2020-09-23

**Authors:** Tomislav Šarić, Ivan Župan, Serena Aceto, Grazia Villari, Dušan Palić, Gionata De Vico, Francesca Carella

**Affiliations:** 1 Department of Ecology, Agronomy and Aquaculture, University of Zadar, 23 000 Zadar, Croatia; tosaric@unizd.hr (T.Š.); zupan@unizd.hr (I.Ž.); 2 Department of Biology, University of Naples Federico II, 80126 Naples, Italy; serena.aceto@unina.it (S.A.); grazia.villari@unina.it (G.V.); gionata.devico@unina.it (G.D.V.); 3 Chair for Fish Diseases and Fisheries Biology, Faculty of Veterinary Medicine, Ludwig-Maximilians-University Munich, 80539 Munich, Germany; d.palic@fisch.vetmed.uni-muenchen.de

**Keywords:** MMEs, *Mycobacterium*, *Haplosporidium pinnae*, epidemiology

## Abstract

From May to October 2019, multiple mass mortality events (MMEs) of *Pinna nobilis* were observed along Croatian coastline starting from the south-east and rapidly progressing in north-western direction. Time dynamics of the MMEs closely followed general speed and direction patterns of surface sea-currents, advancing approximately 350 km in less than 3 months. Surveillance, clinical evaluation, and sample collection were performed on multiple sites with various degrees of mortality rates. Moribund *P. nobilis* individuals were collected and subjected to pathological, molecular, and microscopical investigation. Affected animals were positive for *Mycobacterium* in 70% of the individuals, and *Haplosporidium pinnae* was present in 58% of the cases. Observed pathological lesions were most severe where concurrent presence of both pathogens was confirmed (in 45.8% of moribund individuals). Moderate to strong lesions were observed in animals positive for *Mycobacterium* only (25% of cases), and lesions were absent or minor to moderate when only *H. pinnae* was confirmed (16% of cases). Considering the rapid and severe spread of the MMEs, the areas less exposed to major sea currents appeared to be at lower risk of pathogen transmission. Surveillance activities along the Croatian coastline identified several *P. nobilis* populations in such “lower risk” areas without apparent mortality or clinical symptoms. Such areas are of particular interest as source of potentially healthy individuals to support active recovery actions.

## 1. Introduction

Mass mortality events (MMEs) are known to occur all over the animal kingdom, and their frequency is likely on the rise for many taxa across the globe [1]. MMEs affecting noble pen shell (*Pinna nobilis* L., 1758) were first described in late 2016 in the Mediterranean Sea in Spain [2]. Initial pathological changes observed in dying specimens were associated with a new parasite species from the *Haplosporidium* genus: *Haplosporidium pinnae* [3]. The MMEs were soon observed throughout Mediterranean basin, along the coasts of France, Greece, Cyprus, Italy, and northern Africa [4,5]. In several *H. pinnae* negative cases observed in Italy, Greece and Spain, severe pathological changes were associated with bacteria of the genus *Mycobacterium* [6,7,8]. Recent studies confirmed co-occurrence of several pathogens in the diseased animals, with *Mycobacterium* sp. being most frequently identified, followed by *H. pinnae*, and in a few cases, *Vibrio* spp. and *Perkinsus* sp., suggesting that exposure to multiple pathogens could increase complexity of disease pathogenesis [9]. By the end of 2018, multiple MMEs with high (close to 100%) mortality rates caused complete disappearance of noble pen shell populations in many areas across Mediterranean [10]. Prior to these events, *P. nobilis* was already considered in need for strict protection in the Annex IV of the Habitat Directive, and endangered due to habitat loss since 1995, as reported in the Barcelona Convention. Severity and frequency of recent MMEs pushed this endemic bivalve population to the brink of extinction and prompted the IUCN to change *P. nobilis* status to “critically endangered” in early 2019 [5].

Published data about *P. nobilis* population in Adriatic Sea offer only limited information about population ecology and genetics, and a recent study on animal health status are available [11,12,13,14,15,16,17]. Fisheries data and marine ecosystem surveys suggest that *P. nobilis* populations in the Adriatic Sea have been in decline, likely due to various anthropogenic and environmental influences such as habitat destruction (reduction of *Posidonia oceanica* meadow areas), illegal fishing, pollution, anchoring damage, invasive species, and climate change [18,19,20]. The reverse trend was observed since early 2000, as regulatory actions addressing endangered species were put in place, and an increase in *P. nobilis* populations along Croatian coastline was noted in multiple reports from Adriatic Sea nationally protected areas [21,22,23].

In the Spring of 2019, the first *P. nobilis* MME was detected in the most southern part of Croatian Adriatic coast. During the following period (June–October), multiple MMEs were reported on the Croatian coastline of Adriatic Sea and by mid-July, an ad hoc surveillance program was set up by the University of Zadar with assistance from LMU Munich and University of Naples Federico II. This was, to our knowledge, the first attempt to directly observe dynamics of MME progression in a previously unaffected marine area and collect relevant information to improve understanding of this phenomenon. By late Summer and early Fall 2019, an increase in mortality was confirmed in multiple locations in central Adriatic, with a tendency of spreading further north. Rapid progression of MMEs from South-east to North-west Adriatic appeared to follow prevalent surface sea currents. Acute clinical disease progression and distribution patterns during geographically distinct MMEs strongly suggested involvement of infectious agents. Therefore, the aim of presented work is to characterise rapid spreading of MMEs in the Adriatic in order to improve our understanding of *P. nobilis* emerging disease epidemiology and pathogenesis.

## 2. Results

### 2.1. Disease Epidemiology and Pathogenesis in the Adriatic Sea

In the period from May to October 2019 mass mortality events (MMEs) have been observed in over 60% of sampling locations (7/11), while apparent mortalities were not detected in four remaining locations (see below and Table 1, Table 2 and Table 3 for details).

**Figure 1:**
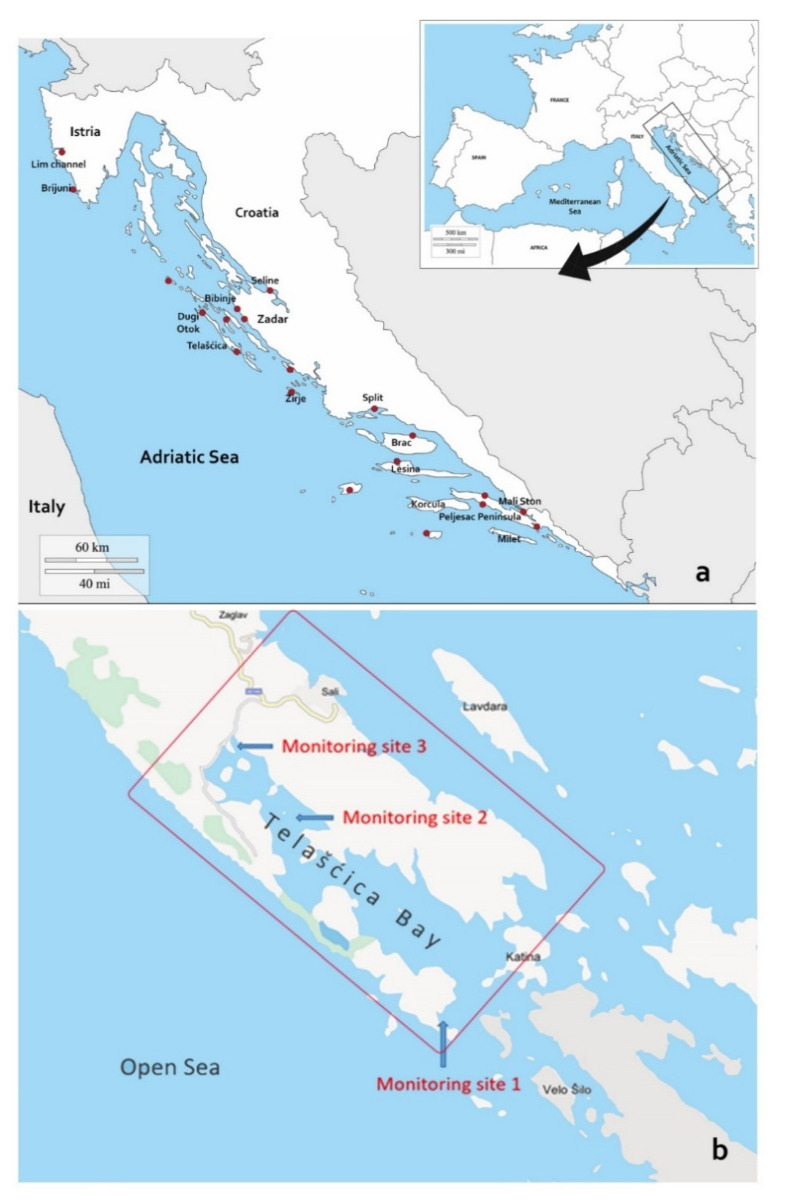
Surveillance sites of *Pinna nobilis* mortality in Croatia from May 2019 to January 2020 (**a**) and details sites at Telašćica Bay (**b**) with the monitoring site 1: Kobiljak Bay; monitoring site 2: Kršovica Bay and monitoring site 3: Magrovica Bay.

Initial mortality event in Croatian waters was observed in May 2019 in the area south of Pelješac peninsula (Figure 1). In the following three months, the MMEs advanced in the general NW direction, with August MME occurring in the NW side of the Dugi Otok on Sakarun beach location, that is over 300 km (straight line) further North-West from the first observation. Mortality rates in various locations ranged between 30–100% depending on the monitoring areas (Figure 1).

**Figure 2:**
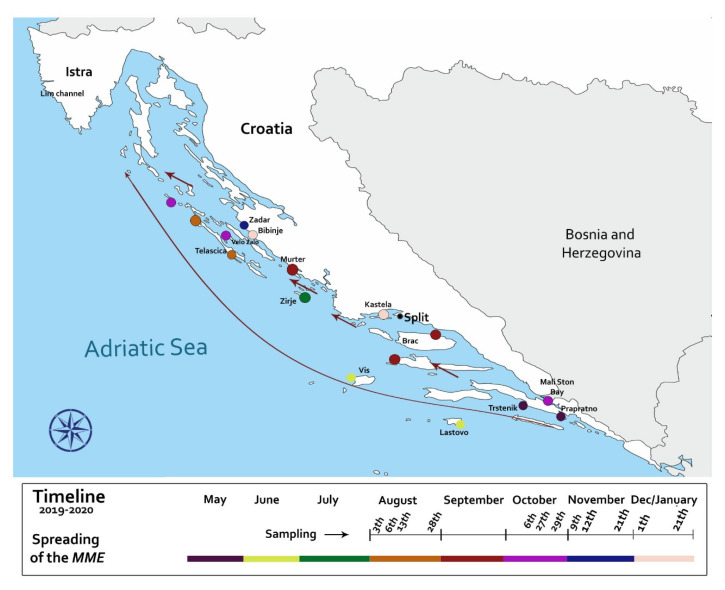
Timeline map of the spread of the MMEs detected from May 2019 to January 2020 in areas along the Croatian coastline and during related sampling periods.

Two distinct patterns of disease progression were observed during the Summer of 2019, one related to animals and another to locations. At the onset of the disease in a given population, larger animals (35–58 cm in length) were displaying clinical symptoms, while smaller/younger individuals (<30 cm total length) appeared unaffected or in better condition than larger ones. Location-wise, *P. nobilis* populations surveyed on outer banks of the offshore islands (Lastovo, Vis) were affected earlier and further away from initial outbreak, while disease outbreaks in populations closer to the continental coastline happened later in the Summer. Furthermore, this location-based pattern was also observed on a smaller scale in Telašćica Bay (see below). Briefly, in this semi-closed bay area approximately 8 km long, an inconsistent mortality rate was observed during August, depending on the location of the monitoring site. In the bay sites that were more exposed to the “open sea”, recorded mortality was 100% (monitoring site 1 and 2; Figure 2), while in the more protected locations (further from the “open sea”), mortality of only 30% was observed (monitoring site 3).

**Figure 3:**
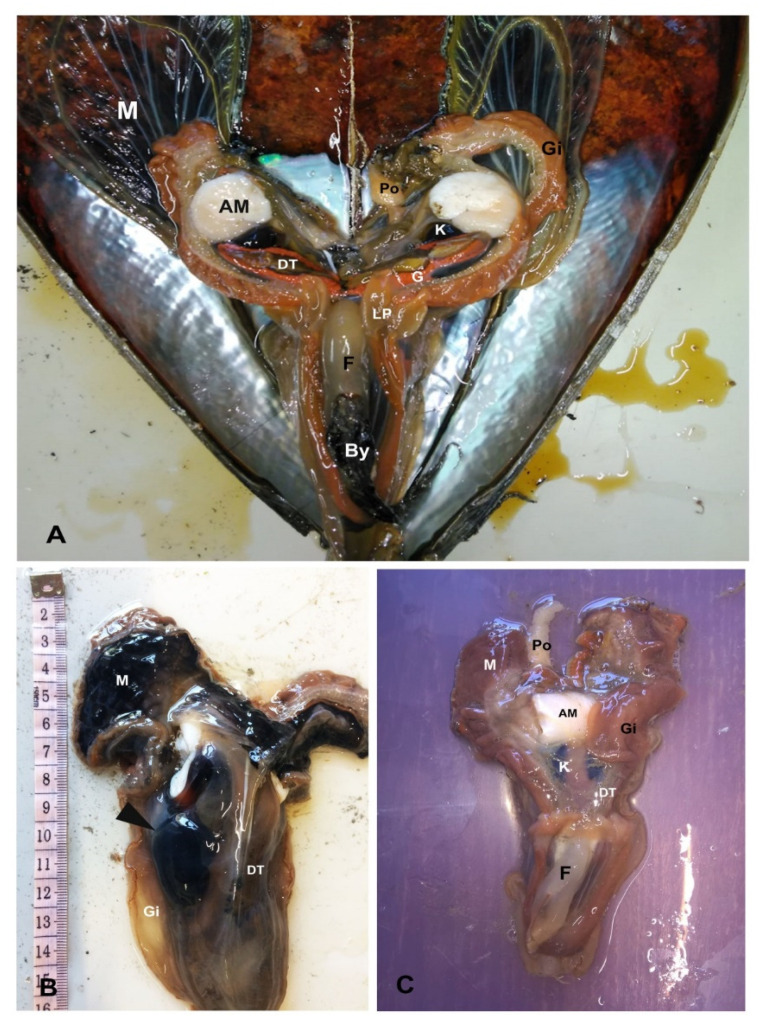
Gross examination of healthy (**A**) and diseased animals (**B**,**C**) from different areas. (**A**) healthy individual showing visible colourful developing gonad (**G**), gills (**Gi**), and deep brown digestive tissue (**DT**); (**B**) diseases specimen from Telašćica Bay shows an evident cyst with liquid content located on the left side of the digestive tissue (**DT**, arrowheads); (**C**) empty watery tissue in a small individual from Mali Ston Bay. **G**: gonad; **AM**: Adductor Muscle; **M**: Mantle; **By**: Byssus; **K**: Kidney; **Gi**: Gill; **G**: Gonad; **Po**: Pallial organ; **F**: Foot; **LP**: Labial Palps.

Post-mortem examination showed that clinical signs related to animal responsiveness were not always representative of animal health status. Animals presenting the promptest response to touch stimuli in situ, resulted positive to different pathogens and lesions after microscope and molecular diagnostics. For example, in apparently healthy specimens collected in Telašćica Bay, a presence of big cysts with a liquid brownish content located at the level of digestive gland was discovered during necropsy (Figure 3). In animals with clinical symptoms, the lesions were represented by various degrees of tissue oedema, watery flashes, and yellowish digestive tissues (Figure 3B,C).

**Figure 4:**
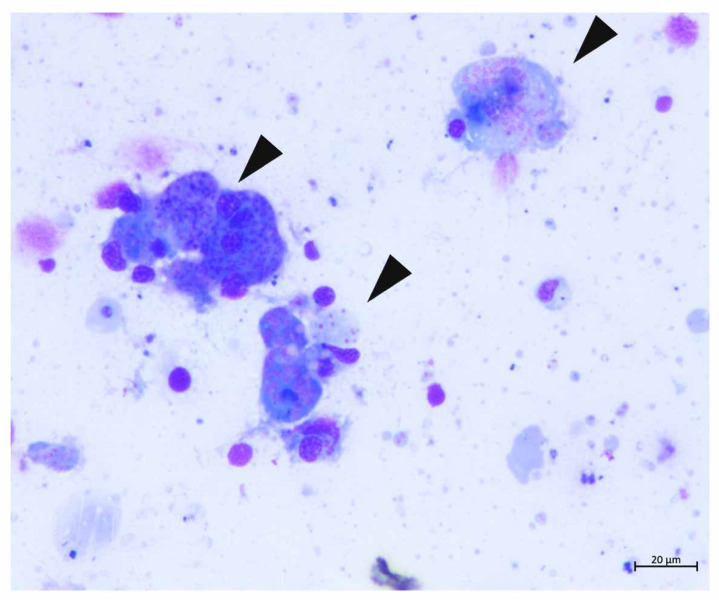
Smear from digestive gland cyst of the individual from Telašćica Bay: different developing plurinucleate pre-sporulation phases of *H. pinnae* parasite. Note multinucleate plasmodium where some cytoplasmic compartmentalisation is distinguished (arrowheads).

Cytology of the collected liquid content revealed different developmental stages of *H. pinnae* as already described by Carella et al. [6] (Figure 4).

**Figure 5:**
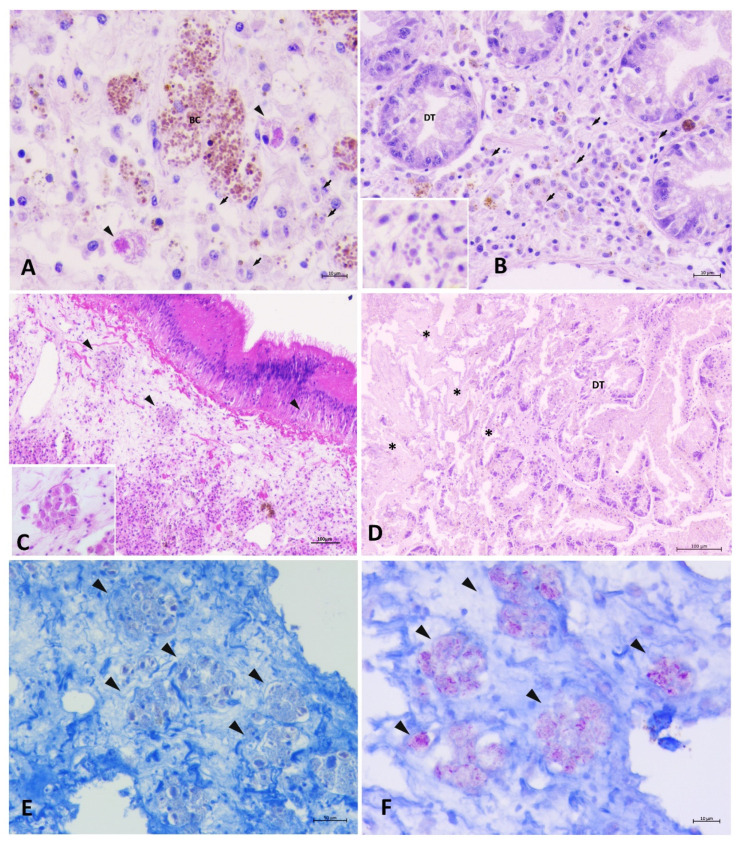
Histopathology of specimens of pen shell *P. nobilis* in August 2019 in Sakarun and Telašćica Bay. (**A**) Co-representation of plasmodial phases of *H. pinnae* (arrows) and *Mycobacterium* (arrowheads) in Sakarun (**BC**: Brown cells); (**B**) diffuse presence of initial phases of *H. pinnae* (arrows) in the digestive gland (**DT**: digestive tubules); (**C**) inflammatory nodules containing *Mycobacterium* (arrowheads) in Sakarun; (**D**) colliquative necrosis of the digestive tubules; (**E**,**F**) Presence of *Mycobacterium* sp. with a grading of infection of score 2 in Telašćica Bay within inflammatory nodules using routine ZN (**E**) and MZN stain (**F**).

**Figure 6:**
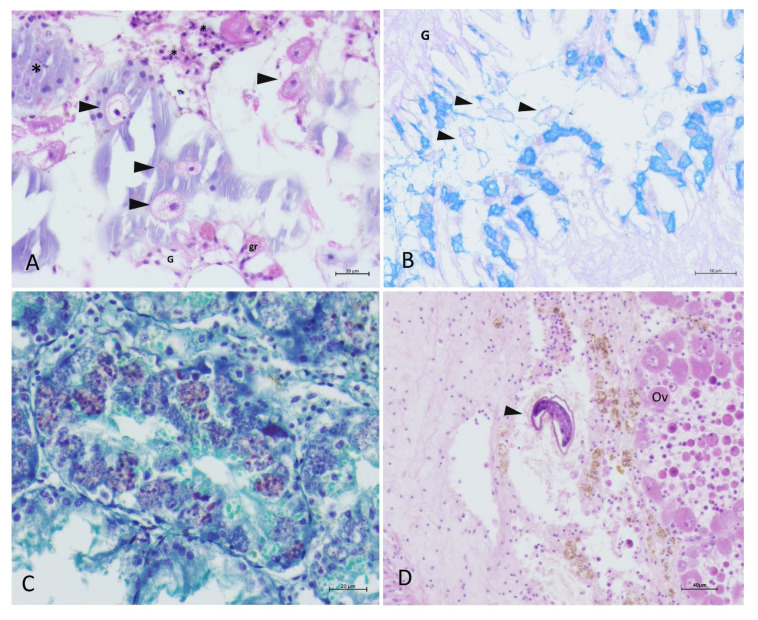
Histopathology pen shell *P. nobilis* in October and November 2019. (**A**,**B**) Presence of ciliates (arrowheads) in the gill (G) accompanied by PAS-BA positive secretions (**B**) and granulocytes (**gr**); (**C**) Advanced phases of development of *H. pinnae* (Masson Trichrome); (**D**) unknown helminth egg in the connective tissue surrounding the gonad (**Ov**: ovocytes) in Kaštela in November 2019.

During August of 2019, surveillance program field surveys started in localities of Sakarun, Seline, Žirje, Telašćica Bay, and for the second time in Bibinje. Histopathological evaluation results are presented in Table 2 and Table 3 and Figure 5 and Figure 6. Detailed description of findings per each sampling site is presented below.

At Sakarun in early August 2019 40% mortality of the *P. nobilis* population was observed. Histopathology revealed the presence of both Mycobacterium and *Haplosporidium* in 100% of the collected moribund specimens (6/6). Plasmodial phases of *H. pinnae* and *Mycobacterium* sp. were concurrently present within haemocytes (Figure 5A). Scarce infiltrative inflammatory response was connected to plasmodial phases of *H. pinnae* in three of the examined samples (Figure 5B). In one case, nodular inflammatory response was observed, linked to both *Mycobacterium* (score 2) (Figure 5C) and *H. pinnae* (score 1). Pathogens were also present in specimens with digestive gland necrosis (Figure 5D). In early August 2019, no mortality was detected in Seline area located further from the open sea in the Velebit Channel.

Animals were negative to both *Mycobacterium* and *H. pinnae*. However, histopathology revealed the presence of inflammatory nodules associated to brown cells hyperplasia in 40% of the animals (2/5 animals). Digestive gland necrosis was also recorded in a single individual, along with the presence of intraepithelial Gram-negative bacteria in the digestive tract. In 60% of the cases, the presence of unidentified ciliated protozoans on gills was also recorded. In August 2019, 90% mortality was observed in the locality of Žirje. The last remaining live specimen in this area was in poor clinical condition when collected for examination. Strong inflammation (infiltrative and nodular) was observed in the mantle and digestive gland cross-sections, but neither *Mycobacterium* nor *H. pinnae* were detected with histopathological or molecular diagnostics.

In Telašćica Bay in August 2019, clinical signs of slow (>10 s) and weak responsiveness were noticed in several animals during field surveillance. Those specimens were considered “sick”, while individuals being more prompt (<5 s) in closing the valves were considered “healthy”. Despite different clinical signs, minor to strong inflammatory lesions linked to the presence of Mycobacterium and *H. pinnae* were observed during necropsy. Further histopathological and molecular diagnostics confirmed both pathogens in both “healthy” and “sick” collected animals. The *Mycobacterium* was present at the level of the connective tissue of mantle as well as in the fibrous capsule surrounding gonad and digestive gland and infiltrating among tubules (score 2). Sometimes, scarcely visible Mycobacteria were observed within immune cell aggregates stained with conventional Ziehl-Neelsen stain, obstructed by the presence of greyish-basophilic material within the cells (Figure 5E). In such cases, the Mycobacterium detection was enhanced through the modified protocol of Ziehl-Neelsen staining (MZN) in all the specimens (Figure 5F). *H. pinnae* was usually observed in an advanced phase of development as characteristic multinucleate cells in the epithelium of a digestive gland tubule with no sporulation.

The location of Bibinje was monitored every two months (June, August and October 2019) without observed mortality. Diagnostic tests in October were negative to both *H. pinnae* and *Mycobacterium* (Table 3). However, just two months later in January 2020, the mortality rate of 80% was observed. Histopathology detected presence of Mycobacterium in 100% (all three) and *Haplosporidium* in 33% (only one) of the collected specimens, accompanied by minor to strong inflammatory response.

In late October 2019, prompted by MMEs advancing toward northern Adriatic Sea, surveillance expanded to locations of Velo Žalo, Lim Channel (Istria), and later in November to Brijuni. At location of Velo Žalo recorded mortality rate was 40%. One of the two sampled specimens presented with mycobacterial infection detected by the light microscopy, but not confirmed with PCR. In the other sample, both *H. pinnae* and *Mycobacterium* were observed at light microscopy, but only *H. pinnae* was detected with PCR. During an initial field visit to Lim Channel, there was no mortality, while histopathology showed the presence of *Mycobacterium* and the absence of *H. pinnae*. It is important to note that in this period (October), the mortality range of 40–60% was recorded in the areas of Mali Ston and Zadar (Central Adriatic) with individuals infected with Mycobacteria in Mali Ston while in Zadar, pathogens were both present. In Mali Ston, the presence of protozoan ciliates in gills was correlated with a strong inflammatory response and associated increase in mucin production in all sampled animals (Figure 6A,B). In November 2019, sampling in Brijuni showed no clinical signs, mortality, lesions, or pathogens.

At the last surveyed site presented in this study, mortality of 95% was recorded in the Bay of Kaštela in January 2020. Both pathogens were present in 60% of the cases as detected with light microscopy, and strong inflammatory lesions and *H. pinnae* in advanced phases of development were observed. The digestive gland epithelium was occupied by parasite sporocysts and developing spores as seen in Figure 6C. Presence of helminths eggs was also observed in the same area linked to a mild inflammatory response (Figure 6D).

### 2.2. Molecular Diagnostic

**Figure 7:**
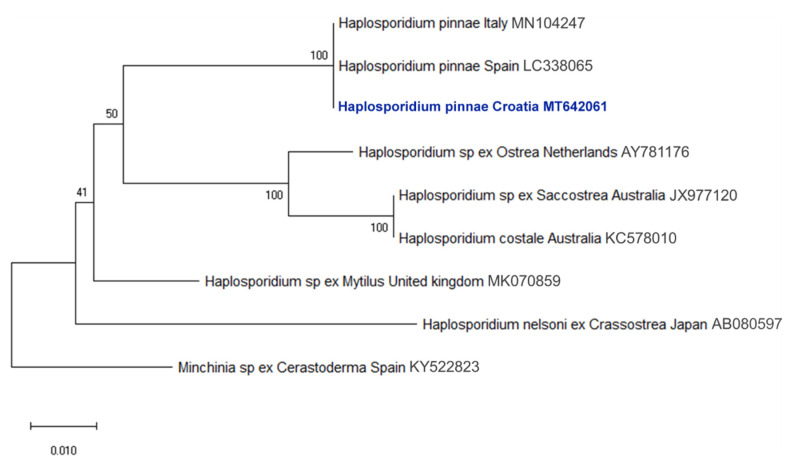
Neighbor-Joining tree of the rRNA sequences of *Haplosporidium* sp. The bootstrap values (1000 replicates) are shown next to the branches. All ambiguous positions were removed for each sequence pair (pairwise deletion option). The accession numbers of the sequences used are reported in the tree. *Minchinia* sp. was used as outgroup.

**Figure 8:**
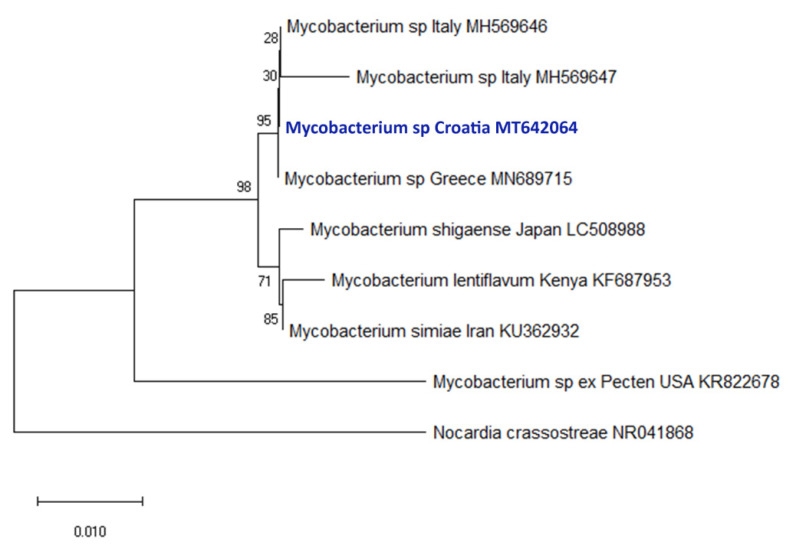
Neighbor-Joining tree of the 16S rRNA sequences of *Mycobacterium* of *P. nobilis*. The bootstrap values (1000 replicates) are shown next to the branches. All ambiguous positions were removed for each sequence pair (pairwise deletion option). The accession numbers of the sequences used are reported into the tree. *Nocardia crassostreae* was used as outgroup.

Molecular analyses confirmed the presence of *H. pinnae* and/or Mycobacteria in all the cases where pathogens were detected during histopathological examinations. The overall concordance between histopathology and PCR in detecting disease-associated pathogens was 75%. The rDNA sequence of *H. pinnae* detected within the Croatian specimens showed 100% nucleotide identity with those infecting *P. nobilis* in Spain and Italy (Figure 7). Similarly, the rRNA sequence of *Mycobacterium* sp. detected within the Croatian specimens showed high similarity (>99%) to those detected in *P. nobilis* from Greece and Italy (Figure 8).

In summary, 91.6% of moribund animal cases were positive for presence of *Mycobacterium* and *H. pinnae* with associated lesions. Mycobacterium was present in 70%, while *H. pinnae* was present in 58% of individuals. Most severe lesions were observed when *Mycobacterium* and *H. pinnae* were present simultaneously (45.8% of cases). However, moderate to strong lesions were noted in the presence of Mycobacterium only (25% of cases), and lesions were absent/minor to moderate in the presence of *H. pinnae* only (16% of cases). In cases with only *H. pinnae*, minor to moderate lesions were observed in the presence of sporulation phases of the parasite, while the lesions were nearly absent in the presence of plasmodia only phase (Table 2).

## 3. Discussion and Conclusions

This is the first study reporting the results of *P. nobilis* disease surveillance program during mass mortality events (MMEs) in a previously unaffected marine area along Croatian coast of the Adriatic Sea during the period from Summer 2019 to Winter 2020. Time and location patterns of MMEs, together with diagnostic data from individuals collected during the MMEs, strongly support the assumption of infectious origin of the disease outbreaks.

Observed timing and locations of the MMEs occurrences during surveillance period, clearly correlate to the direction and speed of main surface currents of the Adriatic Sea (Figure 2) [24,25,26]. It has been previously indicated that corresponding sea currents could be involved in transporting pathogens and/or their still unknown vectors or fomites to promote disease dispersion [27]. Recent studies applied modelling of the sea currents directions and movement to *P. nobilis* MMEs [4]. The surveillance program designed to monitor MMEs in naïve areas of the Croatian coast took into account the major direction and speed of prevailing surface sea currents. This approach allowed us to observe the initiation of MMEs at multiple sites and to be the first to present empirical evidence supporting the role of sea currents in *P. nobilis* disease transmission. In the study, we documented that earlier and more intense outbreaks were occurring in the off-shore islands, to be later followed with less intense mortality events in areas closer to the mainland coastline and in semi-closed bays (Figure 1 and Figure 2).

According to previous reports [8,9], our results support the opinion that both *Mycobacterium* and *Haplosporidium* are involved in the pathogenesis of mass mortality events affecting *P. nobilis* in the Mediterranean Sea. Detection of these two pathogens and presence of associated lesions was observed in 91.6% of the examined sick/moribund animals. The role of the *Mycobacterium* and *H. pinnae* in the pathogenesis of *P. nobilis* MMEs is enforced by absence of mass mortality in the areas where the pathogens have not been detected (see Table 3). Furthermore, the phylogenetic analysis of the pathogens isolated from moribund animals (Figure 7 and Figure 8) showed high similarity of *Mycobacterium* strains (>99%), and *H. pinnae* samples had identical rRNA sequences when compared to previously reported cases from Greece, Spain and Italy [3,6,8]. Therefore, considering the prevalent sea currents at the Strait of Otranto [28] and pathogen genetic similarity/identity, it appears likely that both Mycobacteria and *H. pinnae* have been carried from other areas of the Mediterranean to the Adriatic Sea where they rapidly impacted naïve populations of *P. nobilis*. However, details about transmission patterns, including possible role of other species as reservoirs and/or vectors as well as role of inanimate objects (fomites, such as boats or ballast water) in the epidemiology of the MMEs remain unknown.

As both pathogens can be present in the same animal, and to date, no information about controlled disease challenge following Koch’s postulates is available, it is difficult to determine the relative contribution of each pathogen to disease pathogenesis. The histopathological observation used in the study, along with the scoring method for pathogen infection intensity, is meant to reflect how quickly the disease is progressing to the end stage. In the present study, *Mycobacterium* was detected in 70% of clinically sick and/or moribund specimens, while *H. pinnae* was found in 58%. The significantly increased pathological score/grade (high extent and severity of observed lesions) was detected in specimens with concurrent infection with both *Mycobacterium* and *H. pinnae* (45.8% moribund individuals). However, in the presence of *Mycobacterium* only (25% of cases) the lesions were characterised as moderate to strong, while in the presence of *H. pinnae* alone (16%) the lesions were evaluated as absent/minor to moderate. Furthermore, the *H. pinnae* alone related moderate lesions were concurrent with sporulation phases of the parasite and lesions were absent/minor in the presence of plasmodia. Variable lesion severity during *Haplosporidium* infections indicates that detection of different developmental stages of the parasite could have diagnostic and pathogenic relevance [29,30,31].

Taken together, these results indicate that *Mycobacterium* may be the leading causative in the Adriatic Sea MMEs, as suggested in recent reports [6,8,9]. It is known that pathogenic mycobacteria can undergo a prolonged asymptomatic or latent period, after which disease can be reactivated in a subset of infected hosts [32,33]. Host responses to mycobacterial intracellular presence coincide with avoidance strategies employed by bacterium and include shifts in the host immune response as well as changes in the virulence of the pathogen, leading to periods of remission followed with disease advancements [34,35]. For example, in Istria, during initial sampling from the area without observed mortalities, one individual was positive for mycobacterial infection, and a few weeks later the mortality was recorded in the area. As for *H. pinnae*, Darriba [2] hypothesised that this emerging pathogen could have been a symbiont or facultative pathogen who changed its relationship with the host due to selection pressure from the environment or host conditions [2,36]. Currently, available information suggests that both pathogens need to be considered as major threat to *P. nobilis* at this time.

It should be noted that severe inflammatory lesions were detected in 8.4% of mortalities without confirmed the presence of either of the two pathogens, on sites without pronounced mass mortality. Additionally, during surveillance of the Seline location, strong and diffuse inflammatory lesions were observed in collected specimens without mortalities and presence of neither *Mycobacterium* nor *Haplosporidium*. It was hypothesised recently that other currently unknown causes could be involved in the pathogenesis of this disease [6,9]. It has also been reported that intracellular *Mycobacterium* may be missed by conventional ZN stain [37] and that pathogen detection may be improved by increasing the permeability of the bacterial cells with Triton-X to allow intracellular access of carbolfuchsin-dye. We have applied this approach, together with molecular detection methods to improve the sensitivity and specificity of diagnosis of Mycobacteriosis in *P. nobilis* in this and other studies, in an effort to increase the reliability of negative results in examined individuals.

Massive mortality events of *P. nobilis* are spreading fast and already are affecting all areas of the Mediterranean basin. Only a few natural areas with clinically unaffected populations are currently known [10]. More elaborate field surveillance programs are needed to help identify refugia where populations of *P. nobilis* are thriving, as well as to determine their health status, including presence or absence of currently suspected disease agents. Current surveillance data indicate that such a refuge may be located close to the continental coastline in central Adriatic, in Velebit Channel and nearby locations of Pag Bay, Novigrad Sea, and Karin Sea.

*P. nobilis* is currently critically endangered endemic species with estimated loss of over 90% of the population in the Mediterranean due to rapid spread of this emerging disease. Active actions of disease prevention and control, together with the protection of sanctuary areas, needs to be fully endorsed by the local authorities through national and regional cooperation, involving all stakeholders, including general public and tourism industry.

It is commendable that the critical need for policy supporting control and prevention of emerging disease strategies and programs directed to non-commercial marine species is being recognised by the EU and regional governments. To improve the policy development process, additional information about epidemiology and pathogenesis is needed. Anthropogenic causes are also possible contributing factors to the fragility of the population, as our current understanding of the abiotic component it is still incomplete. It is of high importance to note that collections and culturing of wild *P. nobilis* specimens to be used in larger scale reintroduction programs that are becoming active must be supported with adequate biosecurity, including disease diagnostic, control, and prevention protocols. Indeed, without a specific pathogen or disease-free brood stock, there is an extremely high risk for conservation projects to non-intentionally, albeit deliberately, contribute to spreading of these and possibly other causative agents to the entire Mediterranean basin, and maybe even to other species. Therefore, we recommend that policy makers support collection of further information about disease epidemiology and pathogenesis and encourage data sharing to facilitate monitoring and forecasting as well as development of disease-mapping tools. Such approaches will improve environmental protection, aquatic animal health and the general public awareness in an attempt to prevent the extinction of the noble pen shell.

## 4. Materials and Methods

### 4.1. Sampling Locations and Clinical Disease Surveillance

In the process of developing a surveillance program for Croatian coast of Eastern Adriatic, wider areas with different geographical characteristics were pre-selected for surveillance: it was prudent to include areas that are open to the sea in contrast to the areas closer to inland shoreline, as well as urbanised and protected areas. Next step was to establish communication with citizens and associations for nature protection to engage them in tracking and reporting back with the possible onset of the MMEs in the pre-selected areas. Therefore, final selection of sites for surveillance and monitoring of *P. nobilis* mortality and clinical status was done based on several different elements: (a) geographical location (near/far from the land coast and also in/out of major surface sea currents); (b) presence or absence of on-site mortality as of early July 2019; (c) increased occurrence of empty *P. nobilis* shells (dead animals) as reported by local divers, associations of nature conservation and local authorities (sites with larger populations were considered priority); and (d) accessibility to SCUBA divers or snorkelers (0–20 m average site depth). Surveillance results reported here start from May 2019 to January 2020 (Figure 1). Mortality rate was estimated by counting live and dead individuals within a square area of approximately 10 × 10 m on 20 separate locations (covering geographical area from most southern to most northern Adriatic coast of Croatia). Teams of two SCUBA divers recorded (Canon G16, GoPro Hero 5) and performed visual census (live/dead animal counts), clinical evaluation of individuals (promptness in valve opening/closing and complete/incomplete gap closure) and collected moribund specimens for pathological and molecular diagnostic. Temperature was measured by diving computer SUUNTO D6i, while substrate type was determined according to Bakran-Petricioli [38].

For the purpose of disease surveillance and monitoring during epidemiological investigations, a total of 36 specimens of *P. nobilis* were collected on 11 selected locations from August 2019 to January 2020, with once repeated sampling on Bibinje location (Table 1). Collected individuals with clinical symptoms (moribund or unresponsive/less responsive, >10 s from stimulus to valve closure, and/or weak closure with gap) were presumed diseased, and individuals showing normal responsivity to the touch (<5 s from stimulus to firm valve gap closure) were considered to be clinically healthy. Sample selection criteria were based on targeted surveillance principles as described in OIE Aquatic Code [39], with consideration of the critically endangered population status to collect as few of the animals as possible. Briefly, in order to provide the broadest possible information about disease spreading and role of each pathogen from the least number of animals, we attempted to collect both clinically sick and healthy specimens from areas with ongoing MMEs. We also collected specimens of different sizes when available (<30 and >30 cm) due to observed differences in clinical symptoms. Finally, we collected apparently healthy specimens from areas without noticeable mortality events to investigate for presence or absence of pathogens in populations that were possibly in a pre-clinical phase of the disease outbreak. Sampling was performed under permission from Croatian Ministry of Environmental Protection and Energy (CLASS UP/I-612-07/18-48/145; N°517-05-1-1-18-3 and CLASS UP/I-612-07/19-48/205; N° 517-05-1-1-19-3). Collected bivalves were placed individually in a labelled plastic bag, cooled, and delivered to the laboratory within 24 h from sampling.

### 4.2. Laboratory Diagnostic Examinations

#### 4.2.1. Gross and Microscopical Evaluation

For gross pathology examination, bivalves were measured (maximum length and width of the shells), opened by dissecting the muscle, position and condition of organs was recorded including photo/video of shells, and organs/tissue samples were collected for histopathological and molecular analysis. Cell imprints of digestive gland and haemolymph were collected and stained with May-Grunwald and Gram Stains (Bioptica, Naples, Italy). Tissue samples designated for histopathology (digestive gland, mantle, gills, muscle, gonad, and kidney) were fixed in Davidson’s solution for a week and then stored until analysis in 70% ethanol. Tissue samples were rinsed, embedded in paraffin blocks, and sectioned at 3 μm with a rotary microtome (Bioptica, Naples, Italy). Tissue sections were deparaffinised, stained with haematoxylin and eosin and special stains: V.O.F. (Verde Luz-orange G-acid Fuchsin) [29], Masson Trichrome, Giemsa, Alcian Blu-PAS (Periodic Acid Schiff), Gram [40] and examined by light microscopy (Zeiss, Axioscope A1).

Presence and distribution of *Mycobacterium* and *H. pinnae* were investigated in all collected tissues from each sampled individual and location. For Mycobacteria detection, a modified Ziehl-Neelsen stain (MZN) was performed to increase probability of bacterial detection by increasing the permeability of the bacterial cells as reported for other mycobacterial diseases [37,41]. Briefly, slides were soaked in deionised water, treated with ammonium solution (0.5% in water) for 2 min and dipped in Triton X-100 0.3% (Sigma-Aldrich, Milan, Italy) at room temperature for 30 min. Permeabilised sections were stained with the Carbol-fuchsin ZN solution for 3 h at 60 °C, rinsed with deionised water, decolourised with 3% hydrochloric acid in 95% ethyl alcohol until colour-release stopped and counter-stained with 1% methylene blue for 30 s [37].

During microscopic exam, a semi-quantitative evaluation of pathogen intensity for both *H. pinnae* and *Mycobacterium*, was performed and correlated with the severity of animal lesions. For *H. pinnae* a score was used taking into account developmental stages of the pathogen and its tissue distribution. Each animal was scored by examining two consecutive histological sections. The sections were analysed by two expert pathologists who were blinded to molecular diagnostic to minimise bias. A histological diagnosis was assigned when there was concordance between the pathologists. When two pathologists failed to agree, cases were referred to a third pathologist for arbitration. The scoring was developed as following for *Haplosporidium*: mild infection (score 1): presence of few plasmodia at the level of mantle or digestive tissue; mild to moderate infection (score 2): the parasite is present at digestive gland level and within digestive tubule in the pre sporulation phase within digestive epithelium (until 30% of the DT in a histological section); marked infection (score 3): the parasite is present at the level of digestive tubules epithelium (more than 30% of the DT filled). For *Mycobacterium*, MZN slides were used using similar criteria; mild infection (score 1): few immune cells filled with ZN+ bacteria at mantle level and at digestive tissue capsules; mild to moderate infection (score 2): immune cells aggregates filled with ZN+ bacteria spreading at the connective tissue of mantle as well as in the fibrous capsule of digestive gland, infiltrating tubules and haemolymph vessels; marked infection (score 3): big aggregates of ZN+ bacteria spreading in all the tissues within nodules of haemocytes (digestive gland, mantle and gonad).

#### 4.2.2. DNA Isolation, PCR Diagnosis, and Phylogenetic Analysis

DNA was isolated from different tissues (digestive gland, gill, gonad, muscle), stored in absolute alcohol and performed using the Blood and Tissue Kit (Qiagen, Milan, Italy). DNA quality and quantity were checked with a Nanodrop ND-1000 spectrophotometer (Nanodrop TechnologiesMonza, Italy). In order to determine the presence of *Mycobacterium* sp. and *H. pinnae*, the DNA from every sample was amplified by PCR using primer pairs specific for the 16S rRNA of *Mycobacterium* [42], and for the 18S rRNA of *H. pinnae,* primer pair HPN-F3/HPN-R3, reported by Catanese, [3]. The PCR was performed in 25 μL of reaction volume containing 1 μL of genomic DNA (100 ng/μL), 12.5 μL of GoTaq MasterMix (Promega) at 1× final concentration, 2.5 μL of each primer (10 μM), and 6.5 μL of water. Negative controls without template DNA were also included. The thermal cycle was previously reported by Carella et al. [6] for the *Mycobacterium* sp. and by Catanese et al. [3] for *H. pinnae*. The amplification products were separated using electrophoresis on 2% agarose gel in 1× TAE buffer. After electrophoresis, the fragments of the expected size were gel eluted and directly sequenced (Eurofins Genomics, Milan, Italy). BLASTN analysis was conducted using as queries the nucleotide sequences obtained in the present study. Representative sequences of *H. pinnae* and *Mycobacterium* sp. were then submitted to GenBank (accession numbers MT642061 and MT642064, respectively).

Using ClustalW, the nucleotide sequence of *H. pinnae* and *Mycobacterium* sp. obtained in the present study, were aligned to those of different *Haplosporidium* and *Mycobacterium* species present in GenBank, and selected on the basis of the highest BLASTN score. The Neighbour-Joining trees were obtained from the nucleotide alignments using the Maximum Composite Likelihood model implemented in MEGA X [43] with 1000 bootstrap replicates.

## Figures and Tables

**Table 1:** Monitored areas in Croatia from May 2019 to January 2020.

Date	Location	Coordinates	Water T °C	Substrate Type	Mortality Rate %	Comments
30/05/2019	Pelješac peninsula-Prapratno	42°48′51″ N 17°40′33″ E	18	Sand/Rocks	100	MME-south side of the peninsula, facing towards the open sea
30/05/2019	Pelješac peninsula-Trstenik	42°54′56″ N 17°24′08″ E	18	Sand/Rocks	100	MME-south side of the peninsula, facing towards the open sea
05/06/2019	Pelješac peninsula-Mali ston	42°52′01″ N 17°41′55″ E	18	Mud/Rocks	0	No visible sign of disease-northern side of the peninsula, more protected from the influence of the open sea
05/06/2019	Pelješac peninsula-Janjina	42°55′47″ N 17°26′59″ E	19	Sand/Mud/*Cymodocea nodosa* meadow	0	No visible sign of disease-northern side of the peninsula, more protected from the influence of the open sea
09/06/2019	Bibinje	44°03′30″ N 15°17′08″ E	20	Sand/*C. nodosa* meadow	0	No visible sign of disease
25/06/ 2019	Vis	43°02′18″ N 16°13′59″ E	22	Sand/Rocks	>85	MME
27/06/2019	Lastovo	42°44′41″ N 16°48′41″ E	22	Sand/Rocks	100	MME
15/07/2019	Žirje	43°39′58″ N 15°39′46″ E	23	Sand/*P. oceanica* meadow	>85	MME
10/08/2019	Bibinje	44°03′30″ N 15°17′08″ E	24	Sand/*C. nodosa* meadow	0	No visible sign of disease
03/08/2019	Sakarun (Dugi Otok)	44°07′48″ N 14°52′35″ E	23	Sand/*P. oceanica* meadow	40	Mortality in juveniles is lower; better reaction to stimulus
06/08/2019	Žirje	43°40′01″ N 15°39′38″ E	24	Sand/*P. oceanica* meadow	90	Adults alive with visible signals of disease (valve closure weakness)
13/08/2019	Seline	44°16′20″ N 15°30′38″ E	25	Sand/Rocks	0	No visible sign of disease
28/08/2019	Telašćica Bay (Dugi otok)	43°55′36″ N 15°08′17″ E	25	Sand/Mud/*P. oceanica* meadow	30–100	The mortality rate in locations closer to the open sea was 100% but in closed bays were lower (30%)
22/09/2019	Murter	43°46′42″ N 15°37′17″ E	23	Sand/Rocks	>85	MME
24/09/2019	Hvar	43°13′31″ N 16°32′26″ E	23	Sand/Rocks	30–85	Early signs of MME
25/09/2019	Brač	43°20′57″ N 16°44′24″ E	23	Sand/Mud	30–85	Early signs of MME
06/10/2019	Bibinje	44°03′30″ N 15°17′08″ E	20	Sand/*C. nodosa* meadow	0	No visible sign of disease in moment of sampling
18/10/2019	Premuda	44°20′07″ N 14°35′46″ E	20	Sand/Mud/*P. oceanica* meadow	>85	MME
27/10/2019	Velo Žalo	44°00′15″ N 15°04′1″ E	21	Sand/Rocks/*P. oceanica* meadow	40	Live adult specimens with visible signals of disease
29/10/2019	Lim channel (Istra)	45°07′55″ N 13°43′55″ E	21	Mud/Rocks	0	No visible sign of disease
09/11/2019	Mali Ston Bay	42°52′01″ N 17°41′51″ E	20	Mud/Rocks	60	Mortality in juveniles is lower, better reaction to stimulus
12/11/2019	Zadar	44°08′05″ N 15°12′22″ E	18	Sand/*C. nodosa* meadow	40	Early signs of MME
21/11/2019	Brijuni	44°55′05″ N 13°46′13″ E	18	Mud	0	No visible sign of disease
01/01/2020	Bibinje	44°03′30″ N 15°17′08″ E	13	Sand/*C. nodosa* meadow	80	In period of two months mortality rate reach 80%
21/01/2020	Kaštela	43°33′01″ N 16°21′36″ E	13	Sand/*C. nodosa* meadow	95	MME

**Table 2:** Recorded lesions, pathogens diagnostics and related infection grading in *P. nobilis* over the sampling period between August 2019–January 2020 where MMEs were present with relative percentage of mortalities.

Sample Date (day/month/yr)	Area	Animal Shell Length (cm)	Animal Mortality	Pathogens Diagnostic		Recorded Lesions
				Histopathology Myco/Haplo	PCR Myco/Haplo	
03/08/2019	Sakarun	31	40%	+/+	+/+	Visible plasmodial phases of *H. pinnae* (**score 1**) and presence of *Mycobacterium* within single immune cells infiltrate digestive tissue (**score 1**);
03/08/2019	Sakarun	52		−/+	−/+	Visible plasmodial phases of *H. pinnae* (**score 1**) and presence of *Mycobacterium* within single and immune cells aggregated within digestive tissue (**score 2**), areas of digestive glad necrosis and nodulation. *Mycobacterium* and *H. pinnae* presence within hemolymph vessels.
03/08/2019	Sakarun	19		+ /+	+/+	Visible plasmodial phases of *H. pinnae* (**score 1**) and presence of *Mycobacterium* within single immune cells infiltrate digestive tissue (**score 1**);
03/08/2019	Sakarun	47		−/+	n.p. *	Few plasmodial phases of *H. pinnae* at mantle level (**score 1**); no other lesions visible
03/08/2019	Sakarun	35		−/+	+/+	No lesions visible
03/08/2019	Sakarun	32		+/+	+/+	Scarce inflammatory response; *H. pinnae* (**score 1**); *Mycobacterium* (**score 1**);
06/8/2019	Žirje	54	95%	−/−	−/−	Strong inflammation (infiltrative and nodular) at mantle and digestive gland
28/08/2019	Telašćica	54	65%	+/+	+/+	Strong infiltrative inflammation; *H. pinnae* (**score 2**); *Mycobacterium* (**score 1**);
28/08/2019	Telašćica	49		+/+	+/+	Strong infiltrative inflammation; presence of inflammatory nodules at digestive apparatus and connective tissue level *H. pinnae* (**score 2**); *Mycobacterium* (**score 2**);
28/08/2019	Telašćica	41		+/+	+/+	Light infiltrative inflammation; *H. pinnae* (**score 1**); *Mycobacterium* (**score 1**);
28/08/2019	Telašćica	23		+/+	+/+	Light infiltrative inflammation; *H. pinnae* (**score 2**); *Mycobacterium* (**score 1**);
27/10/2019	Velo Žalo	46	40%	+/−	−/−	No visible lesions; *Mycobacterium* (**score 1**);
27/10/2019	Velo Žalo	53		+/+	−/+	Sporulation phases of *H. pinnae* (**score 3**) and inflammatory nodules filled with *Mycobacterium* in the interstitium of digestive tissue and at fibrous capsule of digestive gland (**score 2**).
09/11/2019	Mali Ston	58	60%	+/−	+/−	Gill inflammation, acid mucin production with presence of ciliates; *Mycobacterium* (**score 2**)
09/11/2019	Mali Ston	55		+/−	+/−	Gill inflammation, acid mucin production with presence of ciliates *Mycobacterium* (**score 2**)
09/11/2019	Mali Ston	57		+/−	+/−	Gill inflammation, acid mucin production with presence of ciliates *Mycobacterium* (**score 1**)
12/11/2019	Zadar	58	40%	−/+	+/−	Light infiltrative inflammation digestive tissue; *H. pinnae* (**score 2**)
01/01/2020	Bibinje	55	80%	+/−	+/+	Strong infiltrative inflammation; *Mycobacterium* (**score 1**);
01/01/2020	Bibinje	47		+/+	+/+	Strong infiltrative and nodular inflammation; *H. pinnae* (**score 2**); *Mycobacterium* (**score 2**);
01/01/020	Bibinje	40		+/−	+/+	Strong infiltrative and nodular; *Mycobacterium* (**score 2**);
21/01/2020	Kaštela	49	95%	+/+	+/+	Strong infiltrative inflammation; *H. pinnae* (**score 2**); *Mycobacterium* (**score 1**);
21/01/2020	Kaštela	38		+/+	+/+	Strong infiltrative inflammation; *H. pinnae* (**score 1**); *Mycobacterium* (**score 1**);
21/01/2020	Kaštela	30		−/−	−/+	Strong infiltrative inflammation; *H. pinnae* (**score 2**); *Mycobacterium* (**score 2**);

* n.p. samples not present.

**Table 3:** Recorded lesions and pathogens diagnostic in *P. nobilis* specimens over the sampling period between August 2019–January 2020 in areas where MMEs where not recorded.

Sample Date (day/month/yr)	Area	Animal Shell Length (cm)	Animal Mortality	Pathogens Diagnostic Histo M/H	Pathogens Diagnostic PCR M/H	Lesions
13/08/2019	Seline	58	0%	−/−	−/−	
13/08/2019	Seline	48		−/−	−/−	Digestive Inflammatory nodules with Browns cells; eosinophilic granulocytes degranulation at mantle level; gill ciliates with light inflammation
13/08/2019	Seline	43		−/−	−/−	light digestive glad necrosis; Bacteria intraepithelial digestive tract; gill ciliates
13/08/2019	Seline	25		−/−	−/−	gill ciliates; mucous production; No visible lesions
13/08/2019	Seline	33		−/−	−/−	gill ciliates; mucous production; No visible lesions
06/10/2019	Bibinje	22	0%	−/−	−/−	No visible lesions
06/10/2019	Bibinje	43		−/−	−/−	No visible lesions
06/10/2019	Bibinje	44		−/−	−/−	No visible lesions
29/10/2019	Istra	47	0%	+/−	+/−	
29/10/2019	Istra	50		−/−	−/−	No visible lesions
29/10/2019	Istra	55		−/−	+/−	No visible lesions
21/11/2019	Brijuni	60	0%	−/−	−/−	No visible lesions
21/11/2019	Brijuni	55		−/−	−/−	No visible lesions
21/11/2019	Brijuni	53		−/−	−/−	No visible lesions
